# Thermoconductive Thermosetting Composites Based on Boron Nitride Fillers and Thiol-Epoxy Matrices

**DOI:** 10.3390/polym10030277

**Published:** 2018-03-07

**Authors:** Isaac Isarn, Xavier Ramis, Francesc Ferrando, Angels Serra

**Affiliations:** 1 Department of Mechanical Engineering, Universitat Rovira i Virgili, C/Av. Països Catalans, 26, 43007 Tarragona, Spain; isaac.isarn@urv.cat (I.I.); f.ferrando@urv.cat (F.F.); 2 Thermodynamics Laboratory, ETSEIB Universitat Politècnica de Catalunya, Av. Diagonal 647, 08028 Barcelona, Spain; ramis@mmt.upc.edu; 3 Department of Analytical and Organic Chemistry, Universitat Rovira i Virgili, C/Marcel·lí Domingo s/n, 43007 Tarragona, Spain

**Keywords:** cycloaliphatic epoxy resin, composites, thermal conductivity, boron nitride, thiol-epoxy

## Abstract

In this work, the effect of the addition of boron nitride (BN) fillers in a thiol-cycloaliphatic epoxy formulation has been investigated. Calorimetric studies put into evidence that the kinetics of the curing has been scarcely affected and that the addition of particles does not affect the final structure of the network. Rheologic studies have shown the increase in the viscoelastic properties on adding the filler and allow the percolation threshold to be calculated, which was found to be 35.5%. The use of BN agglomerates of bigger size increases notably the viscosity of the formulation. Glass transition temperatures are not affected by the filler added, but Young’s modulus and hardness have been notably enhanced. Thermal conductivity of the composites prepared shows a linear increase with the proportion of BN particle sheets added, reaching a maximum of 0.97 W/K·m. The addition of 80 μm agglomerates, allowed to increase this value until 1.75 W/K·m.

## 1. Introduction

Nowadays, electronic and electrical industries have an increasing need to dissipate the heat of devices, which is produced by the Joule effect. This leads to a continuous demand of thermal conductive coatings and adhesives, with high electrical insulation capability. This demand is originated by the constant miniaturization, integration and functionalization of electronics and the appearance of new applications such as flexible electronics, light emitting diodes, etc. In this sense, heat management is of special interest in electronic components since they can be deserved for greater power output, improved efficiency and lengthening of half-life time and prevention of premature failures of devices [1]. These kinds of thermally conductive polymers find also usage in other applications like aerospace industry, heat exchangers and corrosion-resistant coatings and therefore the research in these materials is in constant development [2]. 

Thermal energy is defined by the existence of microscopic vibrations of particles. The temperature, describing the state of a body, is a physical property quantifying those microscopic thermal vibrations of the particles. Heat is directly related to the thermal conductivity (TC), and has been defined as the thermal energy transfer from a specific point to its surroundings due to the temperature gradient [3]. Thus, temperature is produced by particles vibration and heat evaluates how much of this energy is transferred, how fast and in what direction.

Although epoxy resins are extremely valuable materials in coatings and adhesion applications, thermal conductivity of these polymer resins is in the low range from 0.1 to 0.3 W/m·K. The addition of inorganic filler particles into the epoxy material can significantly improve the TC and can also affect the mechanical properties of the composite. However, this constitutes the easiest way to reach the aimed technological goals such as heat dissipation [4,5,6]. Among inorganic fillers, hexagonal boron nitride (BN) is structurally analogous to graphite and has similar thermal conductivity [7,8]. However, for potential electric/electronic applications, BN composites have several advantages over those based on graphene, because of BN is a non-electrically conductor [9]. 

It has been reported that the filler type, loading level, filler size, and filler shape have a strong influence on the thermal conductivity of polymer composites. Creating a continuous filler network is the key point to reach high TC in composite structures. Network formation, usually takes place at high filler loading levels and it is related to the percolation threshold that can be calculated by rheological measurements. It should be noticed that a too high filler content worsens the processability and mechanical properties and increase unnecessarily costs and densities. 

The interaction between filler and polymer matrix is also important in terms of TC enhancement [10]. Phonons are the responsible of heat transmission in amorphous polymers. Because of the mismatches between BN surfaces and the polymer, the interface will result in phonon scattering and hinder the heat transfer. Therefore, improving polymer-filler interfacial interaction can increase the overall composite TC substantially [11]. Several authors reported the modification of BN nanosheets [12,13]. Hexagonal BN particles have a smooth plate-like shape with no available surface functional groups for chemical bonding, but BN particles have hydroxyl and amino groups at the edge planes. Using a simple sol–gel process by reaction with a functional silane compound a higher adhesion between particles and polymeric matrix can be reached with a notable enhancement in TC [13,14].

Recently, Hutchinson et al. [15] reported a notable increase in TC in thiol-epoxy materials filled with BN, without the need of functionalization of the particles. In fact, the thermal conductivities of thiol-epoxy materials were superior to those of the epoxy cured with Jeffamine, which was attributed to a better interface interaction between particles and matrix. They also reported that the increase in TC with filler content was also quicker in thiol-epoxy systems, although high proportions of filler could not be investigated because of the bad workability of the mixture caused by the high viscosity.

Taking into account the improvement in TC of thiol-epoxy materials and the need of a low viscosity of the reactive mixture, we proposed in the present work the use of a cycloaliphatic epoxy resin (ECC) with a commercial tetrathiol (PETMP) as the starting reactive mixture, filled with different proportions of BN filler. The curing of cycloaliphatic resin with thiols in the presence of a tertiary amine, acting as a base, has been previously developed in our research group [16]. The reactive mixture has a low viscosity, which allows adding a high content of BN to the formulations and the materials obtained have a good transparency. As demonstrated in previous publications, this curing system is very versatile since the curing rate and properties of the materials can be tailored by changing the type of amine and the epoxy and thiol structures, respectively [17,18]. Moreover, the polycondensation type polymerization mechanism allows the preparation of more homogeneous networks than those obtained by cationic polyetherification of cycloaliphatic epoxy resins [19]. In that work, the low viscosity of the cycloaliphatic epoxy resin allowed us to reach a 800% of TC enhancement by adding a 40% of unmodified BN as the filler.

Gaska et al. [4] reported that larger particle sizes as agglomerates can lead to higher TC values, according to that, we also try in the present work to improve thermal conductivity by increasing the size of the BN particles. However, the addition of larger particles can negatively affect other properties, like the mechanical performance or viscosity of the formulation. 

The effect of the BN content and the increase in the size of the BN particles on the curing kinetics, viscosities, rheological behavior and gelation phenomena is reported in the present paper together with the thermal and mechanical characterization of the composites obtained. 

## 2. Materials and Methods

### 2.1. Materials

3,4-Epoxy cyclohexylmethyl 3,4-epoxycyclohexane carboxylate (ECC) (ERL-421D, EEW = 126.15 g/epoxyeq) was provided by Dow Chemical Company (Midland, MI, USA). 4-(*N*,*N*-dimethylamino)pyridine (DMAP) was used as initiator, grinded before use and provided by Fluka Analytical (Neu-Ulm, Germany). Pentaerythritol tetrakis (3-mercaptopropionate) (PETMP) (ETW = 122.165 g/thioleq) was purchased by Sigma-Aldrich (Darmstadt, Germany) and used without further purification. Platelets of hexagonal boron nitride (BN) were supplied by ESK Ceramics GmbH (Kempten, Germany), TPC 006, with an average particle size of 6 µm and an agglomerate of 80 µm average, PCTL5MHF, was supplied by Saint-Gobain (Valley Forge, PA, USA) and both were used as received.

### 2.2. Sample Preparation 

The neat mixture was prepared by mixing stoichiometric proportions of ECC and PETMP and adding 1 phr (parts per hundred of resin) of initiator. For composite samples, the required amount of BN was added in wt. % to the neat formulation before curing. The mixtures were mechanically stirred until homogeneity was reached. Finally, the samples were poured onto aluminum molds and cured at 120 °C during 1 h, followed by a post-curing at 150 °C for another 1 h and a final step at 200 °C for half an hour.

### 2.3. Characterization Techniques

To analyze the curing evolution of the epoxy system, a differential scanning calorimeter (DSC) Mettler DSC-821e (Mettler Toledo, Columbus, OH, USA) was used. The device was calibrated using an indium standard (heat flow calibration) and an indium-lead-zinc standard (temperature calibration). Samples of about 5–10 mg were essayed in aluminum pans with a pierced lid in N_2_ atmosphere with a gas flow of 100 mL/min. The scans were performed in the temperature range of 30 to 250 °C with a heating rate of 10 K/min. Curing enthalpies (Δ*h*) of the different samples were calculated by integration of the calorimetric signal. Glass transition temperatures (*T*_g_) of cured samples were evaluated by a second scan as the temperature of the half-way point of the jump in the heat capacity curve. The estimated error was considered to be ±1 °C.

Thermal stability of neat and composite materials was evaluated by thermogravimetric analysis (TGA), using a Mettler-Toledo TGA/DSC 1 Star system (Mettler Toledo). Experiments were performed under N_2_ atmosphere (flux 50 mL/min). Pieces of cured samples of 5–10 mg were degraded between 30 and 600 °C at a heating rate of 10 K/min.

Dynamic mechanical thermal analyses (DMTA) were performed by employing a TA Instruments DMA Q800 device (TA Instruments, New Castle, DE, USA). Samples were isothermally cured in an aluminum mold at 120 °C for 1 h, then at 150 °C for 1 h and finally post-curing at 200 °C for 30 min. Prismatic rectangular samples (15 × 5.0 × 2.3 mm^3^) were tested in 3-point bending mode at a heating rate of 3 K/min in the temperature range from 35 to 125 °C, with a frequency of 1 Hz and oscillation amplitude of 0.1% of sample deformation. The Young’s moduli (*E*) were determined at 30 °C by using a force ramp at a constant rate, 1 N/min, never exceeding 0.25% of deformation to be sure that only elasticity was evaluated. The slope between 0.1% and 0.2% of deformation was taken. *E* was calculated from the slope of the load deflection curve according to the following equation:

(1) $$E=\frac{L^{3}m}{4bt^{3}}$$

where *E* is the elastic modulus of the sample (MPa), *L* is the support span (mm), *b* and *t* are the width and the thickness, respectively, of the sample tested (mm) and *m* is the gradient of the slope in the linear region (N/mm).

Thermomechanical analyses (TMA) were performed on a Mettler TMA40 thermomechanical device (Mettler Toledo). Thermosetting samples (9 × 9 × 2.3 mm^3^) were supported by the clamp and one silica disc to uniformly distribute the force and heated at 5 °C/min from 32 up to 120 °C by application of a minimum force of 0.01 N, to not distort the results. Two heating scans were performed, being the first to erase the thermal history and the second to determine the thermal expansion coefficients (CTEs), below and above the *T*_g_. They were calculated according to the following equation:

(2) CTE=1L0·dLdT=1L0·dLdtdTdt

where *L* is the sample thickness, *L*_0_ the initial length, *t* the time, *T* the temperature and *dT*/*dt* the heating rate.

Surface fractures were examined by using a FEI Quanta 600 environmental scanning electron microscope (ESEM, FEI Company, Hillsboro, OR, USA) that allows collecting electron micrographs at 20 kV and low vacuum mode of uncoated specimens with low electron conductivity. A working distance (WD) of ca. 10 mm was used. 

Microindentation Knoop hardness was evaluated by using a Wilson Wolpert 401 MAV apparatus according to ASTM D1474-13 (Wolpert Wilson Instruments, Aachen, Germany). A minimum of 20 determinations were made for each material with a confidence level of 95%. The Knoop microindentation hardness (KHN) was calculated by using the equation:

(3) $$\mathrm{KHN}=\frac{L}{A_{P}}=\frac{L}{l^{2}C_{P}}$$

where *L* is the load applied to the indenter (0.025 Kg), *A*_P_ is the projected area of indentation in mm^2^, *C*_P_ is the indenter constant (7.028 × 10^−2^) relating *l*^2^ to *A*_P_.

Rheometric experiments were done in parallel aluminum plates (geometry of 25 mm Ø) mode by using a TA AR G2 rheometer (TA Instruments, New Castle, DE, USA), with electrical heated plates (EHP). Viscoelastic characteristics, *G*′ (shear elastic modulus) and *G*″ (viscous modulus), were determined with a constant deformation in the linear viscoelasticity range for each formulation, obtained from constant *G*′ in a strain sweep experiment at 1 Hz, at 30 °C. The curing was followed at 85 °C to determine gel point and conversion at gelation. Gel time was taken as the point where tan δ is independent of frequency [20]. The conversion at the gelation (*x*_gel_) was determined by stopping the rheology experiment when gelation occurred and the sample was quenched in liquid N_2_. Then, the remaining enthalpy was evaluated by a dynamic DSC experiment at 10 K/min. The degree of conversion in the gelation was calculated according to the following equation:

(4) $$x_{\mathrm{gel}}=1-\frac{\Delta h_{g}}{\Delta h_{T}}$$

where Δ*h*_g_ is the heat released up of gelled samples, obtained by integration of the calorimetric curve, and Δ*h*_T_ is the heat associated with the complete curing.

The volumetric content of BN of the different materials prepared was calculated taking into account the densities of the composites determined by means of a liquid pycnometer. The densities of pure BN were taken from the data sheet published by the commercial source company.

Thermal conductivity was determined using the Transient Hot Bridge method by a THB 100 device from Linseis Messgeräte GmbH (Selb, Germany). A HTP G 9161 sensor with a 3 × 3 mm^2^ of area was used. The sensor was calibrated with poly(methylmethacrylate) (PMMA), borosilicate crown glass, marble, Ti-Al alloy and titanium. Two equal rectangular samples, perfectly polished, with size of 12 × 12 × 2.3 mm^3^ were placed at both faces of the sensor. Because of the small size of sensor, the side effects can be neglected. Measuring times of 100 s with a current of 10 mA were applied. Five measures were taken for each material.

## 3. Results and Discussion

### 3.1. Study of the Curing Process

Our research team reported for the first time the thermal curing of biscycloaliphatic epoxy compounds by thiols in the presence of tertiary amines to form new thiol-epoxy thermosets [16]. In our previous publication, we showed that among other tertiary amine catalysts, DMAP was the most suitable to reach the controlled curing of those stoichiometric systems by this thiol-epoxy click reaction. In diglycidylether of Bisphenol A (DGEBA) resins, Hutchinson et al. [15] observed that the reaction kinetics of a thiol-epoxy system was affected by the amount of BN filler added to the formulation, showing an unexpected trend, firstly increasing the curing rate and then retarding it as the filler content increases, without affecting the final cured epoxy network structure. The variations in the reaction kinetics were attributed to an improvement of the interface forces between particles and matrix as a consequence of a Lewis acid-base interaction which finally led to a notable enhancement in the thermal conductivity [15].

**Scheme 1:**
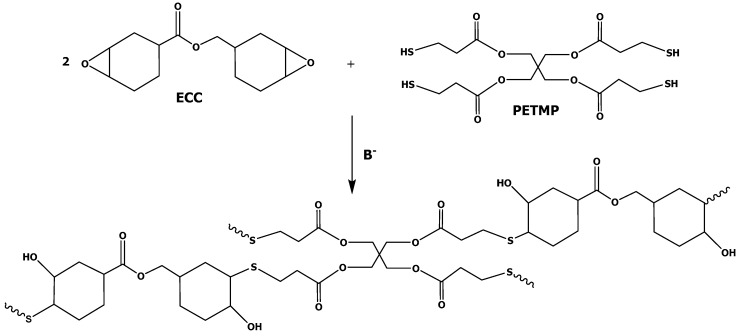
Chemical structures of the monomers used and the network formed during the curing process.

The lower viscosity of cycloaliphatic epoxies in front of DGEBA resins opens the possibility to increase the BN content in the formulation and that led us to start the study of a new BN-filled thiol epoxy system. In this study, we have used 1 phr of DMAP in a stoichiometric formulation of ECC/PETMP with different amounts of BN (10–40 wt. %) of 6 µm average. With the aim to corroborate that the size of the particles plays an important role in the TC we have also prepared a composite with greater particles of BN (80 µm agglomerates). In Scheme 1 the chemical structures of the monomers selected and the network formed are represented.

**Figure 1:**
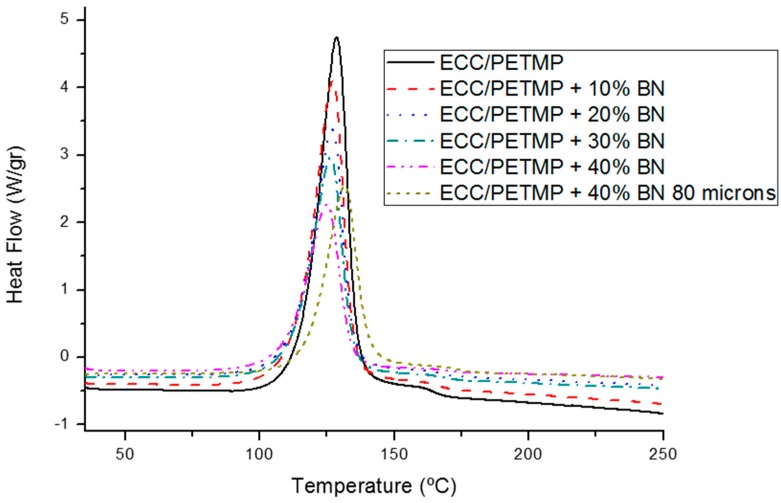
DSC curves showing the effect of the addition of different proportions of BN in wt. % to the formulation.

Figure 1 and Table 1 collect the calorimetric curves and the main data extracted from the curing study.

As we can see, on increasing the proportion of BN in the formulation no much effect is observed, only a slight shift of the maximum of the curing exotherm to lower temperatures and a decrease in the height of the curve, indicating a reduction in the curing rate of the system. However, the addition of the filler with bigger particle size provokes a greater effect, since a displacement of the maximum of the exotherm of 7 °C in reference to the formulation with the same proportion of 6 μm BN is clearly detected. In previous studies of our group in the preparation of BN composites by the cationic homopolymerization of epoxy resins [19,21] we observed much variation in the kinetics of DGEBA matrices than in ECC. As reported, the addition of BN to thiol-DGEBA resins led also to kinetics influence [15]. All these results seem to suggest that DGEBA resin systems are likely to interact better with BN. From the values of the table, it can be seen that the addition of particles does not affect the final structure of the network. This is explained, because of the heat evolved during the dynamic calorimetric scans and the glass transition temperatures (*T*_g_) determined remain practically constant for all the formulations, with a slight decrease in the enthalpy released at the highest proportion of BN, probably due to topological restrictions in curing. 

### 3.2. Rheological Study of the BN Formulations

Any material that cannot be classified as purely elastic or as viscous has a viscoelastic behavior. Polymeric systems, especially those with two or more components, do not obey the Newton’s law of viscosity and present different phenomena since they are considered structured fluids. The design of many industrial processing operations requires taking into account several of these phenomena [22], since their behaviour is generally dictated by the interactions among the components.

**Figure 2:**
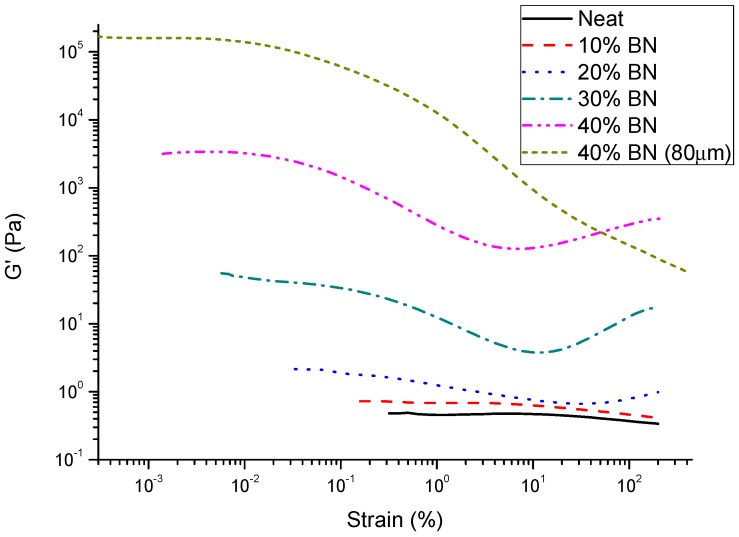
Plot of log *G*′ versus log % strain in oscillatory experiment (1 Hz) of uncured formulations.

The study of filled uncured formulations is recommended to be done by oscillatory experiments and must be performed in the material’s linearity region (LVR) to determine the viscoelastic properties. Thus, a good initial step is to measure the storage and loss moduli (*G*′, *G*″) dependence with the strain amplitude. Figure 2 represents *G*′ (more sensitive than *G*″) versus percentage of strain applied at 30 °C for mixtures without base catalyst to prevent any reaction. It can be observed how the unfilled formulation presents an almost constant storage modulus in all the strain range tested, which means that it has a Newtonian behavior. With the increasing filler content, the Newtonian plateau is shifted to lower strains and became shorter as observed in previous studies [19,21]. However, there is a significant difference at high deformations in the more filled formulations when comparing with these previous studies: it is an increase of the *G*′ with a shoulder shape. Some materials show this behavior due to structure reorganization as the result of the applied deformation, as reported by Laun in polystyrene-ethylacrylate latex particles in water [23]. In our case, the fact that the only difference is the presence of thiol monomer in the mixture, could mean that there was an interaction of thiols with the BN particles. In contrast, the agglomerates only present the typical shear thinning of systems loaded with particles, almost in the frequency range tested, and the plateau is moved even to lower strain. According to the results, the strain was fixed in the LVR for each mixture to perform the oscillatory sweep tests, measuring parameters as function of frequency (*ω*). 

**Figure 3:**
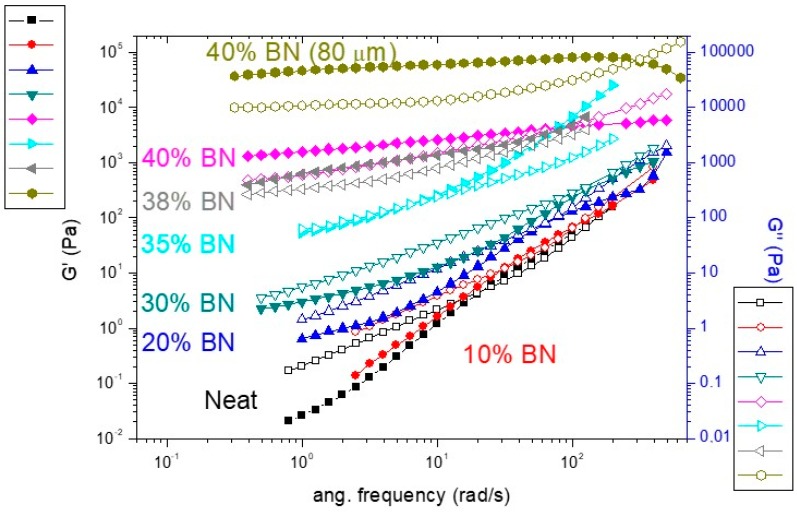
Plots of *G*′ (filled symbols) and *G*″ (open symbols) against *ω* for all the formulations at 30 °C.

To reach good thermal conductivities and to go deeply in the knowledge of our filled system it is interesting to determine the viscoelastic percolation threshold. Percolation is the point in which the particles contact and create a network structure, and the percolation threshold is the minimum filler content in the matrix that produces this network. At this filler percentage a notable change in thermal conductivities and some other properties, can be in principle being observed. To determine this value we have represented *G*′ (elastic property) and *G*″ (viscous property) curves as function of frequency (Figure 3) for all the mixtures studied. We have also included two new mixtures (35 and 38 wt. %) to determine the percolation value more accurately. 

First, it can be seen in the figure that the effect of BN particles in the mixture on *G*′ and *G*″ is not significant at high frequencies (Rouse dynamic region). However, in the region related to reptation dynamics, at low frequencies, the effect is quite important [24]. Moreover, how it was expected, unfilled resin greatly follows the linear viscoelastic rule [25] (*G*′∝*ω*^2^ and *G*″∝*ω*^1^) at low frequencies, but with the increasing content of BN, the slopes continuously decline until the maximum concentration of filler added (40 wt. %), where *G*′ is practically constant on varying the frequency which means that percolation threshold is overpassed.

Above the percolation threshold, the point at which an interconnected network of particles through the whole material is formed, the behavior of the mixtures should obey the scaling law relation at a fixed frequency, that can be used to determine the rheological percolation [22,26]:

(5) $$G^{\prime }\propto {\left(m-m_{c}\right)}^{\beta }$$

where *G*′ is the storage modulus, *m* is the mass fraction of BN composites, *m_c_* is the mass fraction at the rheological percolation and β is the critical exponent. The threshold was calculated to be 35.5 wt. % and the critical exponent 2.4 at 1 rad/s.

It is worth noting that the BN percentage in the percolation threshold is much higher in this system than in the previous study based on BN composites with cationically homopolymerized epoxy matrices (14.4% for ECC and 6.9% for DGEBA) [19,21]. For this reason, two stablished criteria were utilized to confirm the proportion of BN at the percolation calculated previously.

Numerous studies take as valid the criterium for percolation of *G*′~*ω*^0.5^ in the terminal region in small amplitude oscillatory shear (SAOS) experiments [24,27,28]. The slope in *G*′ versus frequency reach a value of 0.5 between formulations with a filler content of 35% and 38% (see Table 2), agreeing this range with the previous calculations. 

Finally, the second criterium employed is an interpretation of Rouse-like behavior: at percolation *G*′ and *G*″ at low frequencies become equal (see Figure 3) [24]. Then, the rheological response at the percolation threshold must show the transition from liquid-like behavior (*G*″ > *G*′) to a solid-like behavior (*G*′ > *G*″). This change is also observed in Figure 3 between the formulations with 35 and 38 wt. % of BN. 

Comparing the rheological behavior of these systems with those previously reported, it is possible to predict a better application as a coating with the same filler loading.

From a practical point of view, it is quite important to know about the gelation phenomenon produced during curing. Gelation occurs when soluble reactants are irreversibly transformed into a three dimensional, infusible and insoluble network. At this point, the system loses its ability to flow and therefore it must be avoided during industrial processing before the shaping of the final material. The polycondensation mechanism in the present thiol-epoxy system must obey the Flory equation and the conversion in the gelation should depend only on the functionality of the monomeric compounds involved in the curing process. Thus, large differences are not expected if BN particles do not play a role in the network formation (although they can be reactive by the presence of reactive groups in the edges of BN sheets). Table 2 shows the data obtained from the gelation studies by rheological measurements at 85 °C. Since filler contents of 35% and 38% were only added to calculate percolation thresholds, the gelation data of these formulations have not been evaluated.

As we can see, there is an increasing trend, although small, in gelation time on increasing the BN proportion. This behavior contrasts with the observed in previous studies on polymeric systems with particle additions [21,29]. This delay to reach the gelation could be attributed to the steric hindrance caused by the BN particles in the formation of the three-dimensional network structure. Moreover, as expected, the conversions at the gelation are kept practically constant for all the mixtures, which confirms that BN particles only act as filler and that the ending groups in the edges of the BN sheets does not participate in the curing. The results obtained in the gelation studies are interesting from the point of view of the application, since the addition of particles allows increasing the pot life of the reactive mixture and does not reduce the conversion at the gelation, in contrast with the results reported in our previous study [21]. In the previous work, the cationic ring-opening homopolymerization mechanism of curing led to a reduction in the conversion at the gelation and to a shorter gelation time. It is important to note that after gelation the material loses its mobility and stresses and small defects could appear because of the shrinkage produced. These problems will be reduced when gelation occurs at higher conversion. The gel point of formulation prepared with 80 µm agglomerates was not evaluated by this technique because of the lack of comparison with other proportions.

### 3.3. Thermal and Mechanical Characterization of BN Composites

**Figure 4:**
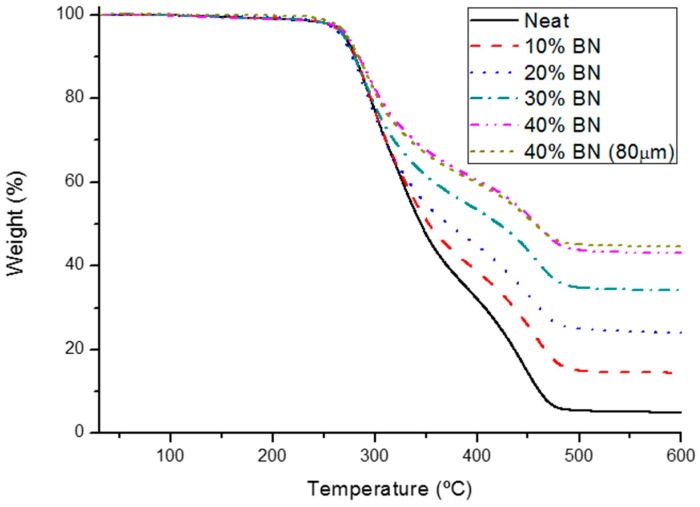
Degradation curves of thermosets obtained by TGA under inert atmosphere at 10 K/min.

Thermogravimetric analysis is the most powerful tool to characterize the thermal stability of the polymeric materials once cured. Figure 4 represents the degradation curves under inert atmosphere.

All the curves show a similar shape, only differentiated in the final residue that results in accordance with the quantity of filler added and a slight increase of the temperature of maximum degradation rate on increasing the BN content in the material (see Table 3). Thus, it can be considered that the degradation mechanism is not affected by the presence of BN, since the network structure of the matrix does not change. In the curves, it can be observed two degradation steps because of the presence of ester groups, both in PETMP and in ECC. The lowest temperature degradation step is related to the decomposition of these ester groups by a β-elimination process that leads to the breakage of the network structure at lower temperatures, and the second one to the scission of the other bonds that occurs simultaneously. It was found in a previous study that the addition of BN to a homopolymerized DGEBA matrix did not significantly affect the thermal stability of composites [21] but in contrast, the temperature of initial degradation suffered an increase of 70 °C on adding a 40% of BN to the cationic homopolymerized ECC matrix [19]. The change in the particle size does not produce great significant changes in the thermal stability behavior of the composites, but only a slight enhancement in the initial degradation temperature and char yield.

Dimensional stability is an important issue when epoxy resins are applied as coatings on any surface, usually metals or ceramics, with a CTE lower than polymers. Oscillating temperature changes can produce premature failures such as separation, blistering, delamination, etc., because of the internal stresses produced by the disparity in their thermal expansion coefficients. To reduce that difference will be beneficial for coating materials to prevent failures and it is know that the addition of BN ceramic particles must positively affect this characteristic [30]. Table 3 presents the CTE values obtained by TMA in the vitreous and in the rubbery state. In the glassy state, there is not a significant change until the value reached for the sample with a 30% BN with a great reduction at 40%, which is the maximum concentration achieved in the composite. This important reduction in CTE between 30 and 40 wt. % of BN could be related to the percolation achieved between these proportions that could lead to a restricted expansion. It has been reported [31] that filler size is an important factor influencing the CTE of composites and that small particles can function effectively to lower the CTE of composites. However, the contrary effect was observed in the present study and the lower CTE was obtained by adding 80 μm BN agglomerates. In the rubbery state, where the matrix is completely relaxed, the diminution is significant above 20 wt. %. Contrary to what observed in the glassy state, there is no difference with the use of bigger agglomerates.

Thermomechanical analysis was performed with DMTA. The filler play the role of matrix reinforcement conferring to material better mechanical performance. Table 4 reports the most important information extracted from the study.

As we can see, Young’s modulus gradually increases with the proportion of particles added. The composite with 40 wt. % of BN shows an improvement higher than 140% in rigidity compared to unfilled material. This is thanks to the anisotropic shape of the BN sheets and to the larger specific surface of these particles. As opposed, the spherical shape of the agglomerates leads to a different behavior when they act as reinforcement. Thus, with agglomerates, the enhancement in Young’s modulus is about 74% compared with the neat resin.

**Figure 5:**
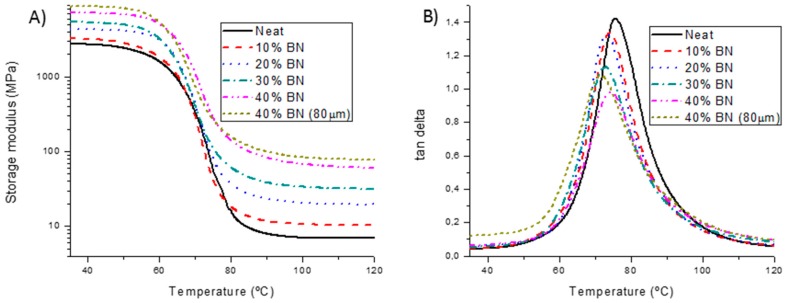
Variation of storage modulus (**A**) and tan δ (**B**) against temperature of the different materials prepared.

Figure 5A shows the variation in the storage modulus with the temperature for the different filler proportions. As we can see, *E*’ is closely related with the filler content because the applied stress is transferred from the polymeric matrix to the BN particles, that have an inherent high modulus. Moreover, the storage modulus in the rubbery state reaches a higher value in the composite obtained with the big agglomerates. This is because in the rubber state, the movement of larger particles is restricted by the other adjacent, while small particles have more freedom of movement and therefore, the material is softer and more deformable.

In the representation of tan δ against temperature shown in Figure 5B we can see that all the composites filled with BN sheets have a quite similar shape and a similar temperature of the maximum (see Table 4) and not much differences were observed in the curves obtained with the composite with agglomerates. The fact that tan δ peak temperatures are not affected by the BN content seems to confirm that no important interactions between filler and matrix exist. This behavior is contrary to that observed previously by us for homopolymerized ECC matrices [19], with enhancement in tan δ values of more than 25 °C. The area of the tan δ peak, which can be associated to the damping characteristics, is decreasing with the increasing amount of filler according to the lower polymer content that can be relaxed. The peak broadens on increasing the BN content, which can be related to the increasing inhomogeneity of the material.

**Figure 6:**
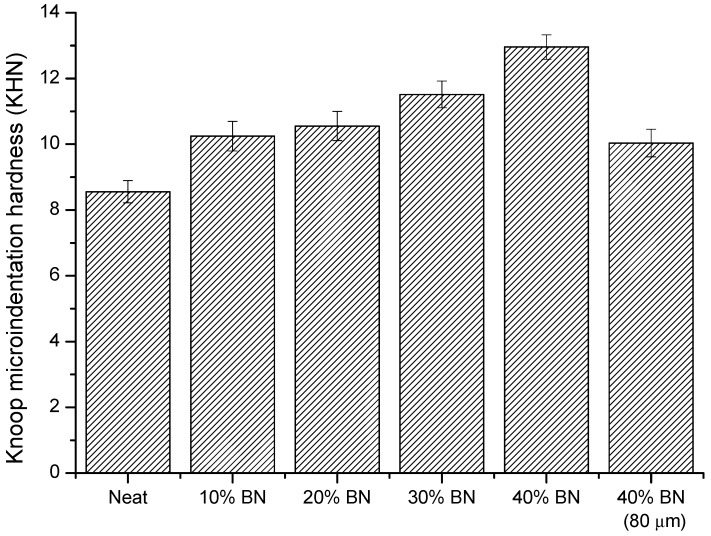
Dependence of the microindentation hardness of ECC/thiol thermosets with different weight percentages of BN.

Since hardness is a desired property for resistance and durability of coatings we have rated how the addition of BN to the neat material affects it. In Figure 6 the Knoop hardness has been represented for all the materials prepared. The increase of BN proportion in the composite leads to an increasing tendency of this characteristic and the maximum is achieved at 40% of BN content. As occurs in the Young’s modulus behavior the addition of agglomerate particles of BN worsens hardness characteristics, due to their big size and less surface of interaction that leads to a smaller reinforcement effect. When comparing these materials to the ones based on homopolymerized ECC (from 11.7 to 26.6), we can see that the reaction with thiols reduced hardness characteristics, because of their flexibility and to the more open network structure formed. Moreover, the increase in hardness with the proportion of BN is much lower in thiol-epoxy materials (50% in front of 150% at 40 wt. % of BN content) [19]. 

### 3.4. Morphology Inspection of BN Composites

**Figure 7:**
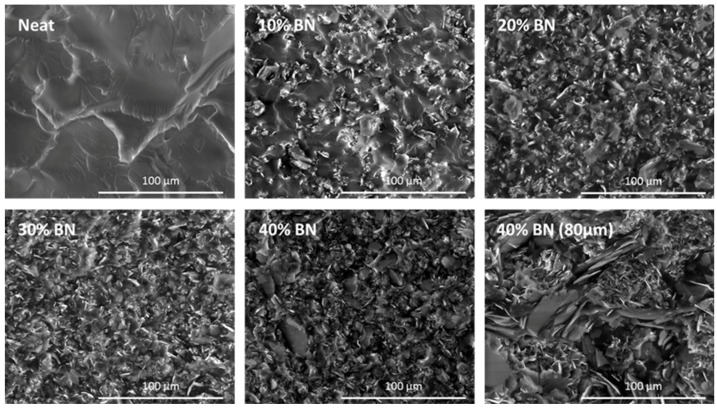
ESEM micrographs of fracture surfaces of the materials prepared at 800 magnifications.

Fracture surfaces were analyzed by ESEM, and the most representative micrographs are collected in Figure 7. Neat polymer has not-linear rupture trajectories with thicker breaks and river-like cracks that accounts for a plastic rupture. On increasing the amount of BN in the formulation, the rupture lines become shorter and more complex due to the action of BN particles that leads to start new paths of breakage. This variation should produce an increase in resilience, the energy absorbed in an impact. If we look at the micrograph of the sample with 10% of BN we can state that it presents a fairly good homogeneity of particle distribution. On increasing the amount of BN the distribution remains homogeneous, but the sample with 40% of BN seems to present a more fragile rupture, that agrees with the percolation achieved in the BN network. As we can see in the corresponding micrograph, the addition of a 40% BN agglomerates leads to a quite inhomogeneous material with a fragile fracture due to the presence of bigger and smaller particles and agglomerates and low adhesion between both organic and inorganic phases.

### 3.5. Thermal Conductivity of BN Composites

**Figure 8:**
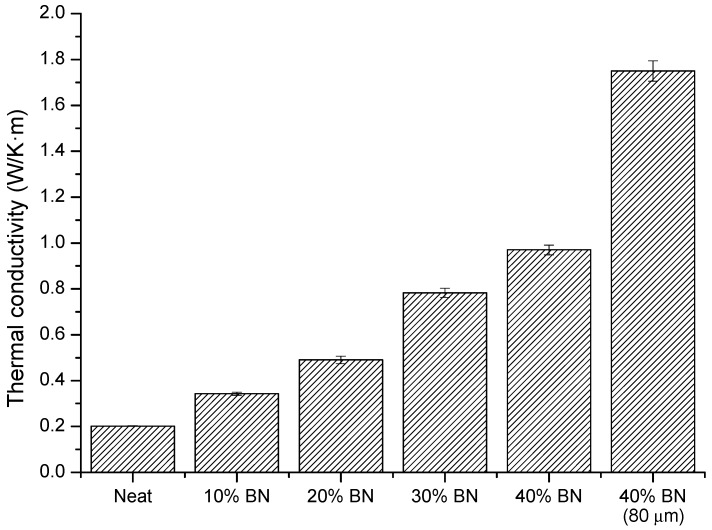
Thermal conductivity of the neat material and composites prepared.

The final goal of this study is to increase thermal conductivities of thiol-epoxy matrices by addition of BN. Thus, the thermal conductivity of the thermosets prepared has been measured and the values are represented in Figure 8.

As we can see, there is a regular improvement in the thermal conductivities with the proportion of BN and at the proportion of 40% an increase of about 400% has been reached. This value of thermal conductivity (0.97 W/K·m) is close to that determined for homopolymerized ECC resins with the same proportion of BN (1.04 W/K·m) [19]. The difference can be attributed to the different particle-matrix interaction in both materials, better in homopolymerized ECC according to the participation of hydroxyl end-groups in the BN in the homopolymerization mechanism. 

The conductivities measured in this study do not reach the values obtained by Hutchinson et al. [15] in DGEBA-thiol systems, which are higher than 2 W/K·m using the same type of BN as filler. However, the values obtained in the present study are similar or even higher than some reported in the literature [13,32,33,34]. 

It is worth noting that the addition of bigger agglomerate particles has led to a notable improvement in this characteristics and the value of 1.75 W/K·m (775% increase) has been reached. The enhanced value can be explained according to that reported by Gaska et al. [4] who reached high values by using bigger particle sizes, because of heat transfer through the polymer matrix is much less efficient than through the crystalline filler. Since the main reason of the low thermal conductivity in polymer composites is the phonon scattering, especially at the interfaces, it is foreseeable that at a determined filler loading the thermal conductivity increases with increasing particle size due to the smaller interfacial area between filler and matrix [31]. In contrast, many authors have reported that nanoparticles produces better enhancements in thermal conductivity [31,35]. This improvement is probably due to an increase of the intrinsic thermal conductivity of fillers, connected to a symmetry-based selection rule that strongly suppresses phonon-phonon scattering in 2D particles [36]. However, the use of nanoparticles enlarges the amount of filler-matrix interfaces and they seem not to be the best choice to obtain high thermal conductivity composites [3]. 

**Figure 9:**
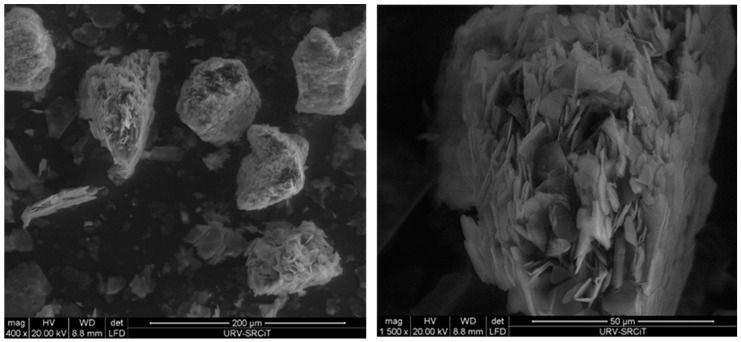
ESEM micrographs of the pure BN agglomerates at 400 (**left**) and 1500 (**right**) magnifications.

In our case, by using 80 μm agglomerates, there is a great dispersion in particle sizes and a big amount of interphases, which can reduce the expected increase in thermal conductivity if the particles were purely crystalline. Figure 9 shows the ESEM micrographs of the pure BN agglomerates.

After curing (see Figure 7) both agglomerates and separated nanosheets can be observed dispersed in the polymeric matrix, in which big and small BN particles are well distributed. It is foreseeable that inside the agglomerates may not have penetrated the resin, subsequently reducing the interaction area in comparison to what happens in the BN sheets.

## 4. Conclusions

Calorimetric studies put into evidence that the kinetics of the curing was scarcely affected and that the addition of particles did not affect the final structure of the network. There is an increasing trend, although small, in gelation time on increasing the BN proportion in the formulation but no differences were observed in the conversion at the gelation, which indicates that BN particles did not play a role in the curing mechanism.

The percolation threshold was calculated by rheometry to be 35.5 wt. % and the critical exponent 2.4 at 1 rad/s.

The thermal stability and the degradation mechanism were not affected by the presence of BN. The addition of BN to the formulation reduced the thermal expansion coefficient of the final materials. Bigger BN particles results advantageous in this reduction.

Young’s modulus gradually increased with the proportion of BN particles added, but the addition of BN agglomerates had a lower reinforcing effect, due to the different shape and size of fillers. The same trend was observed in the hardness enhancement. Glass transition temperature did not varied on increasing the BN proportion, but storage modulus increased due to the reinforcement of particles. On increasing the amount of BN in the material damping characteristics and homogeneity were reduced.

By ESEM inspection, it was possible to see that on increasing the amount of BN in the formulation, the rupture lines became shorter and more complex due to the action of BN particles that led to start new paths of breakage. This variation should produce an increase in resilience, the energy absorbed in an impact.

The addition of BN particles to the thiol-epoxy matrix resulted in a substantial increase in thermal conductivity of about 400% (0.97 W/K·m) at the highest proportion added. This value was highly improved (1.75 W/K·m, 775% increase) if 80 μm agglomerates were added.

## Figures and Tables

**Table 1:** Calorimetric data of formulations ECC/PETMP with several proportions of BN.

BN (wt. %)	*T*_max_^a^ (°C)	Δ*h* ^b^ (J/g)	Δ*h* ^b^ (kJ/ee)	*T*_g_^c^ (°C)
0	127	479	120	58
10	126	436	121	58
20	126	376	118	57
30	125	337	121	57
40	124	272	114	57
40 (80 µm)	131	276	116	58

^a^ Temperature of the maximum of the curing exotherm; ^b^ Enthalpy of the curing process by gram of mixture or by epoxy equivalent; ^c^ Glass transition temperature determined by the second scan by DSC after a dynamic curing.

**Table 2:** Rheological fitting results at 30 °C and gelation data from rheometric monitoring of the curing of the formulations at 85 °C.

BN (wt. %)	*G*′_slope_^a^ (Low Freq.)	*G*″_slope_^a^ (Low Freq.)	*t*_gel_^b^ (Min.)	*x*_gel_^c^ (%)
0	1.81	1.02	16.3	59
10	1.77	1.06	17.8	62
20	1.34	0.99	18.3	60
30	0.62	0.84	18.4	55
35	0.57	0.66	-	-
38	0.36	0.27	-	-
40	0.22	0.24	19.5	55
40 (80 µm)	0.11	0.08	-	-

^a^ Slopes of viscoelastic properties at low frequencies (potential functions in log-log diagrams); ^b^ Gel time determined from the frequency independent crossover of tan δ; ^c^ Determined as the conversion reached by rheometry and DSC test at 10 °C/min.

**Table 3:** Thermal data of composites extracted from TGA and TMA analysis.

BN (wt. %)	BN (vol %)	*T*_2%_^a^ (°C)	Char Yield ^b^ (%)	*CTE*_glass_^c^ (10^−6^·K^−1^)	*CTE*_rubber_^c^ (10^−6^·K^−1^)
0	0	249	5.0	69	195
10	6.0	250	14.4	68	192
20	12.8	249	24.0	66	166
30	20.2	250	34.2	67	157
40	28.2	252	43.0	55	134
40 (80 µm)	27.4	259	44.7	43	135

^a^ Temperature of 2% weight loss determined by TGA in N_2_ at 10 °C/min; ^b^ Char residue at 600 °C; ^c^ Thermal expansion coefficient in the glassy state determined between 38–52 °C and in the rubbery state between 70–90 °C.

**Table 4:** Thermomechanical data of composites varying BN concentration.

BN (wt. %)	Young’s Modulus ^a^ (GPa)	*T*_tan δ_^b^ (°C)	*E’*_rubber_^c^ (MPa)	*Peak*_area_^d^	FWHM ^e^ (°C)
0	2.3	75	6.9	1.37	13.7
10	2.4	74	10.4	1.28	14.6
20	3.6	73	19.6	1.27	14.8
30	4.5	73	31.9	1.13	16.0
40	5.6	74	61.2	0.90	16.8
40 (80 µm)	4.0	71	78.5	0.88	19.3

^a^ Young’s modulus determined with DMTA at 30 °C in a controlled force experiment using three point bending clamp; ^b^ Temperature of maximum of the tan δ peak at 1 Hz; ^c^ Relaxed modulus determined at the *T*_tan δ_+ 40 °C (in the rubbery state; ^d^ Area of tan δ peak between 40 and 120 °C; ^e^ FWHM stands for full width at half maximum.
